# Sulforaphane Inhibits Lipopolysaccharide-Induced Inflammation, Cytotoxicity, Oxidative Stress, and miR-155 Expression and Switches to Mox Phenotype through Activating Extracellular Signal-Regulated Kinase 1/2–Nuclear Factor Erythroid 2-Related Factor 2/Antioxidant Response Element Pathway in Murine Microglial Cells

**DOI:** 10.3389/fimmu.2018.00036

**Published:** 2018-01-23

**Authors:** Erden Eren, Kemal Ugur Tufekci, Kamer Burak Isci, Bora Tastan, Kursad Genc, Sermin Genc

**Affiliations:** ^1^Izmir International Biomedicine and Genome Institute, Dokuz Eylül University, Izmir, Turkey; ^2^Department of Neuroscience, Health Science Institute, Dokuz Eylül University, Izmir, Turkey

**Keywords:** sulforaphane, microglia, lipopolysaccharide, nuclear factor erythroid 2-related factor 2, Mox phenotype, miR-155

## Abstract

Sulforaphane (SFN) is a natural product with cytoprotective, anti-inflammatory, and antioxidant effects. In this study, we evaluated the mechanisms of its effects on lipopolysaccharide (LPS)-induced cell death, inflammation, oxidative stress, and polarization in murine microglia. We found that SFN protects N9 microglial cells upon LPS-induced cell death and suppresses LPS-induced levels of secreted pro-inflammatory cytokines, tumor necrosis factor-alpha, interleukin-1 beta, and interleukin-6. SFN is also a potent inducer of redox sensitive transcription factor, nuclear factor erythroid 2-related factor 2 (Nrf2), which is responsible for the transcription of antioxidant, cytoprotective, and anti-inflammatory genes. SFN induced translocation of Nrf2 to the nucleus *via* extracellular signal-regulated kinase 1/2 (ERK1/2) pathway activation. siRNA-mediated knockdown study showed that the effects of SFN on LPS-induced reactive oxygen species, reactive nitrogen species, and pro-inflammatory cytokine production and cell death are partly Nrf2 dependent. Mox phenotype is a novel microglial phenotype that has roles in oxidative stress responses. Our results suggested that SFN induced the Mox phenotype in murine microglia through Nrf2 pathway. SFN also alleviated LPS-induced expression of inflammatory microRNA, miR-155. Finally, SFN inhibits microglia-mediated neurotoxicity as demonstrated by conditioned medium and co-culture experiments. In conclusion, SFN exerts protective effects on microglia and modulates the microglial activation state.

## Introduction

Microglial cells are the resident macrophages and surveillant cells of the central nervous system (CNS). Under normal physiological conditions, they are found in a resting state and continuously scan their microenvironments to clear cell debris and provide neurotrophic support to maintain the health of surrounding neurons (1). Various exogenous and endogenous immune stimuli activate microglial cells and start innate immune responses. Upon activation, microglia can phagocytose invading pathogens, scavenge amyloid plaques, and remove cell debris (1, 2). Microglial activation may result in different outcomes, which are either beneficial or detrimental, depending on the severity and duration of the stimuli. Perpetual overactivation of microglia causes release of various neurotoxic mediators, including reactive oxygen species (ROS), reactive nitrogen species (RNS), and pro-inflammatory cytokines (3). These mediators result in oxidative stress, neuroinflammation, and mitochondrial dysfunction, thereby leading to loss of surrounding neurons and glial cells. They also promote the activation of neighboring microglial cells and microglial toxicity. Persistent activation of glial cells is also implicated in the pathogenesis of neurodegenerative diseases (4, 5).

Activated microglia can show various phenotypic and functional activation states, namely classical activation (M1) and alternative activation (M2) (6, 7). M1 phenotype accelerates inflammatory response and leads to tissue injury. M1 microglia produces and secretes pro-inflammatory cytokines, including tumor necrosis factor-alpha (TNF-α), interleukin-1 beta (IL-1β), and interleukin-6 (IL-6). Lipopolysaccharide (LPS) is a classical M1 phenotype inducer in microglia. M2 phenotype contributes to resolution of inflammatory responses. IL-4 reduces pro-inflammatory cytokine release as a main M2 polarization stimulus. Arg1, CD206, Fizz1, and Ym1 are most commonly used M2 markers and they contribute not only to inflammation resolution but also to tissue repair (8, 9). In addition, different microglial activation states have also been defined (10). One of them is called Mox phenotype and peripheral macrophages polarized by oxidized phospholipids have recently been identified (11). This phenotype is characterized by a distinct gene expression profile, including upregulation of Ho-1, sulfiredoxin (Srxn-1), glutamate cysteine ligase catalytic subunit (Gclc), and Thioredoxin reductase 1, which are target genes of nuclear factor erythroid 2-related factor 2 (Nrf2) transcription factor. Dynamic and continuous transitions occur among these different activation states, depending on the nature of the environmental stimuli and the phases of different inflammatory responses. As seen in the peripheral immune system, various autoregulatory mechanisms, such as transitions between different activation states, dampen and terminate immune responses (6, 7). When these mechanisms do not work, microglia undergo overactivation and the consequent self-propelling inflammatory responses would require exogenous modulation with various therapeutic agents to dampen them. Recently, such approaches for the modulation of microglial responses have been intensively studied in neurodegenerative diseases. These approaches also include natural products such as phytochemicals (12).

Sulforaphane (SFN) is an isothiocyanate found in cruciferous vegetables (13). SFN is found at highest concentration in broccoli. SFN content of broccoli is 37–75 mg/100 g (14). SFN could be administered, such as broccoli seed powders, broccoli-sprout powder, or capsules. Daily intake of homogenate of fresh broccoli sprout from 22 to 200 g was found safe and effective (15). Bioavaibility of SFN may also be determined by Nrf2 target enzyme activity. Its antioxidant, anti-inflammatory, cytoprotective, and anti-apoptotic effects at non-toxic concentrations have been intensively studied (16, 17). Higher concentrations of SFN exert cytotoxic effect in various cell types. However, these concentrations cannot be achieved through diet or supplement [reviewed in detail in Ref. (15)]. In a number of experimental *in vitro* and *in vivo* studies, SFN exerts neuroprotective and glioprotective effects against neurotoxic agents and LPS (18–21). SFN has anti-inflammatory effects in LPS-induced inflammation in rodent microglia (22, 23). These effects are based on the inhibition of pro-inflammatory transcription factors nuclear factor kappa-light-chain-enhancer of activated B cells (NF-κB) and activator protein 1(AP-1) (23, 24).

Sulforaphane is also a well-known inducer of Nrf2 transcription factor, which transcribes various antioxidant, cytoprotective, and anti-inflammatory genes. Nrf2 is inactive in the cytosol when bound to its inhibitor Kelch-like ECH-associated protein 1 (Keap1). However, as a consequence of increased intracellular ROS, Nrf2 is released from Keap1 and translocates into the nucleus (25). Following translocation to the nucleus, Nrf2 heterodimerizes with small Maf proteins and binds to antioxidant response elements (AREs) found in promoter regions of its target genes, such as Ho-1 and NAD(P)H Quinone oxidoreductase 1 (Nqo1), Srxn1, glutathione S-transferase P (Gstp1), and Gclc (13, 26–28). SFN-activated Nrf2 mediates its antioxidant, cytoprotective, and anti-inflammatory effects (17, 29). However, it is still unclear whether SFN has any Nrf2-dependent effect on microglial activation status and cell death.

Posttranscriptional gene regulatory mechanisms may also contribute to the effects of SFN. MicroRNAs (miRNAs) are 22-nucleotide long members of a small non-coding RNA family, which posttranscriptionally regulate many fundamental cellular processes, such as inflammation, differentiation, and apoptosis (30). Mature miRNA molecules exert their function by binding to 3′-UTR regions of their target mRNA either to cause destabilization or translational repression. MiRNAs also play a role in the brain to fine-tune gene expression for development, neuronal activities, and inflammatory responses (31). Modulation of miRNA by various therapeutic agents could become a novel therapeutic approach for a wide range of human diseases (32). Similarly, SFN may exert anti-inflammatory effects *via* altering miRNA expressions.

Sulforaphane is a good candidate molecule for the treatment of neurodegenerative diseases due to the ability of crossing blood–brain barrier and presence of pleiotropic effects. Therefore, we chose SFN as a protective agent in our study. In the present study, we showed that SFN has modulatory effects on the microglial activation state, which results in a state similar to the gene expression pattern of the Mox phenotype. Furthermore, SFN can inhibit microglial cell death and inflammatory responses through the activation of Nrf2 transcription factor.

## Materials and Methods

### Reagents

Fetal bovine serum (FBS), RPMI 1640 medium, DMEM:F12, l-Glutamine, penicillin/streptomycin, phosphate-buffered saline (PBS), and trypsin/EDTA were purchased from Biochrom (Germany). Lipopolysaccharide (Cat#: L6529, 055:B5), Camptothecin (Cat#: C9911, PubChem CID: 24360), S-Nitroso-*N*-acetyl-dl-penicillamine (SNAP) (Cat#: N3398; PubChem CID: 5231), Nω-Nitro-l-arginine methyl ester hydrochloride (L-NAME) (Cat#: N5751; PubChem CID: 39836), Protoporphyrin IX zinc (II) (Cat#: 282820; PubChem CID: 24857074), and SFN (Cat#: S6317; PubChem CID: 9577379) were purchased from Sigma-Aldrich (USA). All PCR primers and antibodies are listed in Tables S1 and S2 as Supplementary Material in supporting information.

### Cell Culture and Treatments

N9 microglial cells were kindly provided by Dr. Paola Ricciardi-Castagnoli (Cellular Pharmacology Center, Milan, Italy). Cells were cultured in RPMI 1640 supplemented with 10% FBS, 2 mM l-Glutamine, 100 U/ml penicillin and 100 µg/ml streptomycin at 37°C in a humidified atmosphere containing 5% CO_2_. SH-SY5Y cells were maintained in DMEM:F12 supplemented with heat-inactivated FBS (10% v/v), 2 mM l-glutamine, and 100 U/ml penicillin and 100 µg/ml streptomycin at 37°C in 5% CO_2_. Microglial cells were pretreated with SFN for 1 h followed by LPS (100 ng/ml) treatment for 24 h unless otherwise stated.

Primary mouse microglial (PMG) cells were obtained from Sciencell Research Laboratories (CA, USA). PMG cells were isolated from neonate day 2 mouse brain tissues. Purity of PMGs was characterized by OX-42 (CD11b/c) immunofluorescent staining. PMGs were maintained in DMEM:F12 culture medium supplemented with%10 FBS, 2 mM l-Glutamine 100 U/ml penicillin, and 100 µg/ml Streptomycin at 37°C in humidified 5% CO_2_.

Since, SFN may influence several types of cell death, we used different cell death analysis methods, including lactate dehydrogenase (LDH), water-soluble tetrazolium-8 (WST-8), Propidium iodide (PI) staining, Annexin V-FITC/PI, Cell death enzyme-linked immunosorbent assay (ELISA), and Caspase-11 and -3 activities to determine effects of SFN against LPS- induced microglial cell death.

### Cell Viability Assay

Cell viability was evaluated by using WST-8 containing Cell Counting Kit-8 (Sigma Aldrich, USA). Cells were treated with a reagent of interest, and at the end of incubation period, 10 µl of WST-8 solution was added to each well and incubated for 1 h at 37°C. Absorbance was measured at 450 nm with reference wavelength of 630 nm. Cell viability was expressed as a percentage of WST-8 in untreated control cells.

### LDH Assay

Cytotoxicity Detection Kit LDH (Roche, Germany) was used to quantify cell death. After treatment, cell culture media were collected, and LDH activity was quantified according to manufacturer’s protocol. Absorbance was measured at 492 nm (reference wavelength: 630 nm) on a spectrophotometer (Biotek, USA). Cytotoxicity is expressed as a percentage of the total amount of LDH released from lysed cells.

### PI Staining

Propidium iodide staining was used to detect cell death. Briefly, PI (Sigma-Aldrich, USA) (50 µg/ml) was added into media and incubated for 15 min. Stained cells were imaged using Olympus BX50 fluorescent microscope (Japan). PI positive cells were counted using ImageJ 1.51n software and data were presented as percentage of PI positive cells.

### Detection of Apoptosis and Necrosis

Annexin V-FITC/PI (Biovision, USA) staining was used for quantitative detection of apoptosis and necrosis. After treatment, cells were incubated with 5 µl of Annexin V-FITC and PI for 5 min at room temperature according to the manufacturer’s instructions. Stained cells were immediately analyzed by flow cytometer (Becton Dickinson, USA). In each experiment, 1 × 10^4^ cells were analyzed. FlowJo (TreeStar, USA) software was used for data analysis.

### Annexin-V Immunostaining

Cells were stained by Annexin V-FITC apoptosis detection kit I (BD Pharmingen™, USA). After staining procedure, cells were fixed with 4% paraformaldehyde for 20 min, and then stained with DAPI. Stained cells were immediately viewed on an Olympus BX50 fluorescent microscope (Japan).

### Detection of DNA Fragmentation

Intranucleosomal DNA fragmentation was detected by antibody-capture of mono- and oligonucleosomal histone-DNA complexes by Cell Death Detection ELISA kit (Roche, Germany). At the end of 24 h cell incubation with LPS, cell culture media were removed and cells were incubated at room temperature for 30 min with lysis buffer. Aliquots were then transferred to a microtiter plate and a mixture of anti-histone-biotin labeled antibody and anti-DNA peroxidase-conjugated antibody was added. The plate was incubated for 2 h at room temperature. Subsequently, peroxidase substrate was added to each well, and the plate was incubated for 20 min. The absorbance was read at 405 nm (reference wavelength: 490 nm) (Biotek, USA).

### Caspase-3 and Caspase-11-Like Activity Assay

Cellular caspase-3 and caspase-11-like activities were measured with a colorimetric caspase activity assay kit (Invitrogen, USA). Briefly, 200 µg of protein was diluted to 50 µl with lysis buffer, and the lysate was mixed with 50 µl 2× reaction buffer (containing 10 mM dithiothreitol), and caspase substrate peptides Ac-DEVD-pNA for Caspase-3 and Ac-LEHD-pNA for Caspase-11(33). The reaction was performed at 37°C for 2 h. The cleaved para-nitroaniline, with a light emission at 405 nm, was quantified using a spectrophotometer (Biotek, USA).

### ELISA Assays

Tumor necrosis factor-alpha, IL-1β, and IL-6 are pro-inflammatory cytokines that are secreted after an inflammatory stimulus. Their secretion levels in cell culture supernatant were measured by using commercial ELISA kits (eBioscience, Austria) according to the manufacturer’s instructions. The absorbance was determined at 450 nm using a microplate reader VarioSkan Flash (Thermo Scientific, USA).

### Nitrite Assay

Nitrite in the culture medium was measured to assess NO production in microglial cells. Briefly, 50 µl culture medium were mixed with 50 µl Griess reagent (Sigma-Aldrich, USA) and incubated at room temperature for 10 min. Absorbance was measured at 540 nm with ELISA reader (Biotek, USA). Nitrite concentration in each sample was calculated by standard curve using NaNO_2_.

### Intracellular ROS Quantification

Intracellular ROS quantification was determined by 2′,7′-dichlorodihydrofluorescein, acetyl ester (CM-H_2_DCFDA, Invitrogen). First, black 96-well plate was used for seeding cells. Cells were treated with SFN for 6 h and then 100 µl PBS containing 10 µM CM-H_2_DCFDA was added to each well, the plate was incubated for 1 h. Afterward, CM-H_2_DCFDA was removed from the wells, and cells were treated with appropriate stimuli. Fluorescence levels were determined at excitation 492 nm and emission 527 nm on a microplate reader (BioTek, USA).

### Mitochondrial Membrane Integrity

Mitochondrial membrane potential measurement was measured using JC-1 dye. Briefly, cells were stained with 2.5 µg/ml JC-1 (Life Technologies, USA) for 15 min in the dark at room temperature according to manufacturer’s protocol and measured with fluorescence plate reader (Varioskan, ThermoScientific). Furthermore, mitochondrial membrane potential was analyzed by FACS Canto II analyzer using 488 nm laser (Becton Dickinson, USA). Mitochondrial depolarization was shown as decrease in the red/green fluorescence intensity ratio.

### Quantitative RT-PCR

For mRNA quantification, total RNA was isolated using RNeasy mini kit (Qiagen, Germany) according to manufacturer’s instructions. cDNAs were synthesized by Revert-Aid cDNA synthesis kit (ThermoFisher, USA) using 5 µg of total RNA with random hexamer primer and amplified using the mouse primers for target genes (Table S1 in Supplementary Material). For normalization mRNA data, endogenous Glyceraldehyde-3-phosphate dehydrogenase was used.

For miRNA quantification, miRNeasy mini kit (Qiagen, Germany) was used to isolate total RNA and miScript RT II (Qiagen, Germany) kit was used for cDNA synthesis. Real-time qPCR was performed using miScript SYBR Green PCR Kit (Qiagen, Germany) on a Lightcycler^®^ 480 Real-Time PCR System (Roche Diagnostics, Germany). Primers for mature miR-155, miR-146a, miR-223, U6, and SNORD95 were purchased from Qiagen (Valencia, CA, USA). Changes in both mRNA and miRNA levels were analyzed relatively according to comparative threshold cycle (ΔΔC_T_) method.

### *In Situ* Hybridization (ISH)

For ISH analysis, primary microglial cells were seeded into Poly-l-Lysine coated 4-chambered slides at a density of 2 × 10^4^ cells/chamber. After overnight incubation, cells were pretreated with, SFN (5 µM) for 1 h. Following pretreatment, N9 cells were stimulated by 100 ng/ml LPS (Escherichia coli 055:B5; Sigma) for 24 h. At the end of incubation, cells were fixed with 4% paraformaldehyde in PBS for 20 min and washed twice with PBS. Cells were processed as described by Cardoso et al. (34) and hybridized with Cy3 labeled mmu-miR-155 or scrambled probes (Exiqon, Denmark) accordingly. Cells were counterstained with 4′,6-diam idino-2-phenylindole (DAPI). Cells were visualized with Zeiss Epifluorescence microscope equipped with apotome and analyzed with Axiovision software.

### Western Blot

Equal amounts of proteins were loaded and separated with 12% SDS-PAGE and transferred onto polyvinylidene fluoride (PVDF) membranes (Sigma-Aldrich, USA). The membranes were blocked in 5% bovine serum albumin in Tris buffered saline containing % 0.05 Tween-20 (TBS-T) except phosphoproteins and membranes were blocked with 5% milk in TBS-T for phosphoproteins. Then, membranes were incubated overnight 4°C with primary antibodies (Table S2 in Supplementary Material) according to manufacturer’s instructions. Membranes were then washed three times with TBS-T, and then incubated with the horseradish peroxidase (HRP)-conjugated secondary antibodies. The antigen–antibody complex was detected by chemiluminescence using the Supersignal West Dura ECL reagent (Thermo Scientific, USA) and images were captured with Chemi-Smart 5100 (Vilber Lourmat, France). Band densities were analyzed by using ImageJ 1.51n (NIH, USA) and normalized to β-actin or Lamin A/C as loading control.

### NF-κB, AP-1, and Nrf2 Activation Assays

Nuclear extracts were used to quantify DNA binding of the p65 subunit of NF-κB, AP-1 (c-fos and c-Jun) and Nrf2 using TransAM Transcription Factor Assay Kits (Active Motif, USA). After incubation, cells were scraped and nuclear and cytosolic fractions were isolated with Cell fractionation kit (Biovision, USA). Protein concentration was determined using Bicinchoninic Acid Kit for Protein Determination (Santa Cruz, CA, USA) according to manufacturer’s instructions. Briefly, 20 µg of protein per well was added to 96-well plates coated with consensus NF-κB sequence (5-GGGACTTTCC-3), consensus AP-1 sequence (5-TGAGTCA-3), and ARE consensus-binding site (5-GTCACAGTGACTCAGCAGAATCTG-3). After binding of transcription factors to target DNA, wells were washed and primary antibodies and HRP-conjugated secondary antibodies were added, respectively. The plate was washed after the secondary antibody incubation, and then chromogen was added into wells and absorbance at 450 nm was measured with ELISA reader (Biotek, USA).

### Immunofluorescent Staining for Nrf2 and NF-κB

Cells were fixed with 4% Paraformaldehyde in PBS and permeabilized with 0.2% Triton X-100 in PBS for 10 min at room temperature and blocked for 30 min in 5% Normal Donkey Serum (Jackson Immunoresearch, USA) in PBS. Cells were stained with Nrf2 or NF-κB p65 primary antibodies in PBS, containing 2.5% Normal Donkey Serum, for 2 h at room temperature and then Alexafluor-594 conjugated donkey anti-rabbit IgG (Jackson Immunoresearch, USA) for 50 min. Hoechst H33342 (1 µg/ml) was added during the last 20 min to counterstain DNA. Finally, after three washes with PBS, cells were mounted with antifade reagent and observed with BX61 fluorescent microscope (Olympus, Japan).

### Nrf2 siRNA Knockdown

N9 cells were seeded into 96-well plate in antibiotic-free complete RPMI 1640 medium (Gibco, USA). Next day, antibiotic-free RPMI 1640 media with and without 10% serum (Gibco, Life Technologies, USA) containing 100 nM siRNA (Dharmacon, USA) was prepared with Dharmafect transfection reagent (Dharmacon, USA). One-hundred-microliter media were added to each well and were incubated for 24 h. After incubation, medium of each well was replaced with RPMI 1640 containing 1% FBS according to experimental groups. After 24 h incubation, cell viability was measured with WST-8 and NO levels were measured with Griess Reagent. Furthermore, ROS levels were measured using CM-H_2_DCFDA (Life Technologies, USA). Inflammatory cytokine secretions were measured using appropriate ELISA assays (eBioscience, Austria).

### Co-Culture and Conditioned Media Assays

N9 cells were seeded on 6-well plate and after overnight incubation, cells were treated with or without SFN for 1 h, and then treated with LPS for 24 h using DMEM:F12 media. On the treatment day of N9 cells, SH-SY5Y cells were seeded on a 48-well plate. After N9 cell treatment, media were removed and filtered using 0.22 µm filter and filtered conditioned media were added onto SH-SY5Y cells. After 24 h treatment with conditioned media, cell viability was assessed with PI staining. Neurite number and length were measured using ImageJ version 1.51n.

Briefly, N9 cells were plated on upper part of transwell inserts (0.4 µm pore size). Next day, N9 cells were treated with SFN for 1 h and then treated with LPS for 24 h. SH-SY5Y cells were seeded on a separate 24-well plate. After overnight incubation, coculture setup was completed by combining N9 cells in transwell inserts with SH-SY5Y cells. After 24-h incubation period, cell viability of SH-SY5Y cells was assessed with PI staining. Neurite number and length were measured using ImageJ version 1.51n.

### Neurite Outgrowth and Count

Phase contrast images of SH-SY5Y cells were obtained and neurite-bearing cells were counted from selected five fields with identical cell confluency from cell cultures in triplicate. For neurite length measurements, minimum of 30 neurites from each neurite-bearing cell were quantified using ImageJ version 1.51n.

### Data Analysis

Statistical analyses were performed using GraphPad Prism 6.0 (GraphPad Software Inc., CA, USA). Shapiro–Wilk test was used for normality test and if distributions were normal, one-way ANOVA with Bonferroni multiple comparison correction was used to compare the experimental groups. When there were experiments with two independent groups, data were analyzed with Mann–Whitney *U* test. *p* Values <0.05 were considered as statistically significant.

## Results

### SFN Protects N9 Microglial Cells from Cell Death

**Figure 1:**
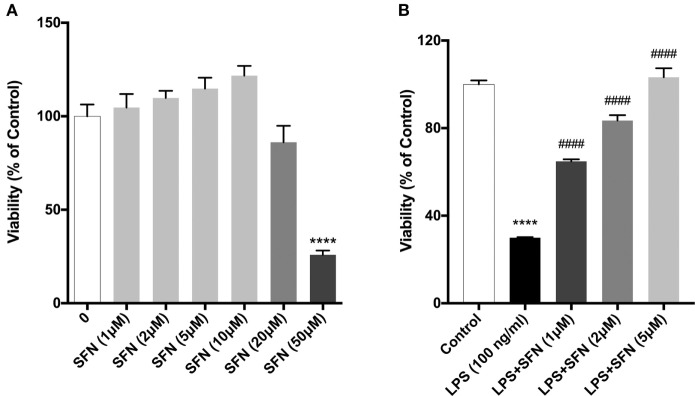
Sulforaphane (SFN) protected N9 cells against LPS-induced cell death. N9 cells were treated with **(A)** increasing concentrations of SFN for 24h, or **(B)** in the presence or absence of SFN (0.5–5µM) for 24h and cell viability were evaluated. Low concentrations of SFN, up to 10 µM, did not affect cell viability. The results are mean ± SEM, *n* = 5 (****p < 0.0001, compared with untreated control. ^####^*p* < 0.0001 compared with LPS treatment).

We evaluated the effects of SFN on cell viability using a WST-8 assay. N9 cells were incubated with increasing concentrations of SFN for 24 h. Higher concentrations of SFN (20 and 50 µM) decreased cell viability (Figure 1A).

We then tested the protective effects of SFN in LPS-treated N9 microglial cells by using WST-8 assay. Treatment of N9 microglial cells with LPS significantly decreased cell viability and pretreatment of SFN increased N9 cell viability in a dose-dependent manner (Figure 1B).

### SFN Prevents LPS-Induced Apoptosis and Necrosis

**Figure 2:**
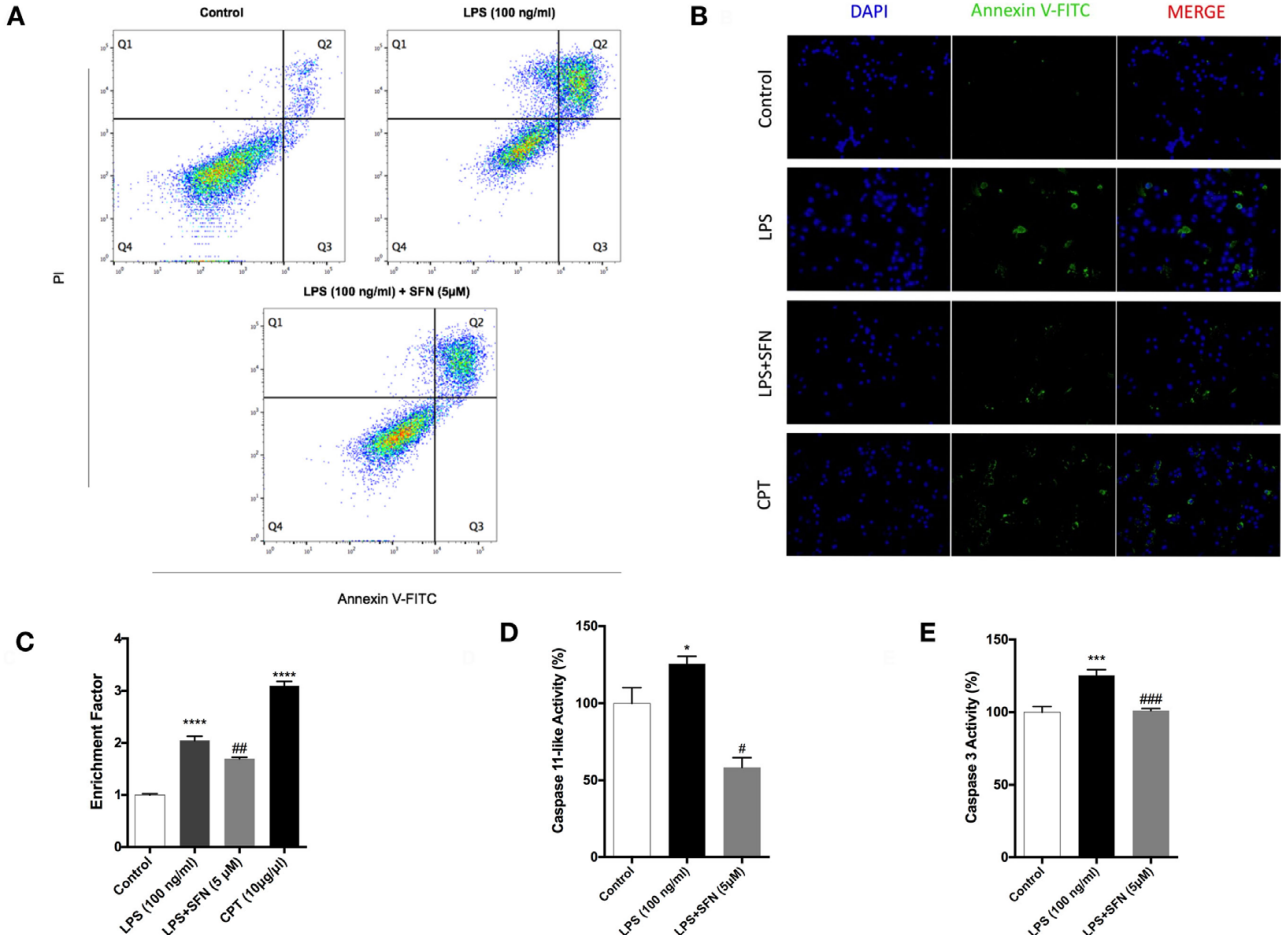
Attenuation of apoptotic and necrotic cell death by sulforaphane (SFN) pretreatment. N9 cells were treated with SFN (5 µM) 1 h prior to lipopolysaccharide (LPS, 100 ng/ml) treatment for 24 h. **(A)** Flow cytometry analysis with Annexin V-FITC/PI were used for apoptotic and necrotic cell death. **(B)** Cells were stained with Annexin V-FITC for visualizing apoptotic cells. **(C)** DNA fragmentation analysis was performed by using cell death enzyme-linked immunosorbent assay (ELISA) assay. Activities of **(D)** caspase-11-like and **(E)** caspase-3 were assessed. The results are mean ± SE, *n* = 5 (*p < 0.05, ***p < 0.001, and ****p < 0.0001 compared with untreated control. ^#^*p* < 0.05, ^##^*p* < 0.01, and ^###^*p* < 0.001 compared with LPS treatment alone).

To analyze the effects of SFN on LPS-induced cell death, we used Annexin V staining. We found that SFN pretreatment suppressed LPS-induced apoptotic cell death (Table 1; Figures 2A,B). SFN pretreatment also reduced LPS-induced necrosis (Annexin V-/PI+) in N9 cells (Table 1; Figure 2A). DNA fragmentation analysis also confirmed that SFN pretreatment reduced LPS-induced apoptosis (Figure 2C). Overall, our findings clearly demonstrated that LPS treatment induced apoptosis and necrosis in microglial cells, and SFN pretreatment attenuated LPS-induced both cell death types.

**Table 1:** Flow cytometry results.

	Q1	*p*-Value	Q2	*p*-Value	Q3	*p*-Value	Q4	*p*-Value
Control	0.90		4.57		92.68		1.86	
Lipopolysaccharide (LPS)	11.85	0.0286	38.48	0.0286	47.80	0.0286	1.86	0.9143
LPS + sulforaphane	2.155	0.0286	31.10	0.1143	64.53	0.0286	2.21	0.0286

### SFN Reduces LPS-Induced Caspase-11 Like and Caspase-3 Activities

Caspase-11 expression is induced by inflammatory stimuli and activates downstream effector caspases, such as caspase-3. We, therefore, tested the possibility that SFN might contain an inhibitory activity on caspase-11 and caspase-3 induction. We found that caspase-11-like activity was 25% higher in cells that were treated with LPS for 24 h (Figure 2D). SFN pretreatment completely inhibited caspase-11-like activity in N9 cells (Figure 2D). Similarly, SFN pretreatment significantly reduced caspase-3 activity, which was increased with LPS treatment for 24 h (Figure 2E).

### SFN Decreases Pro-inflammatory Cytokine Production in N9 Microglial Cells

**Figure 3:**
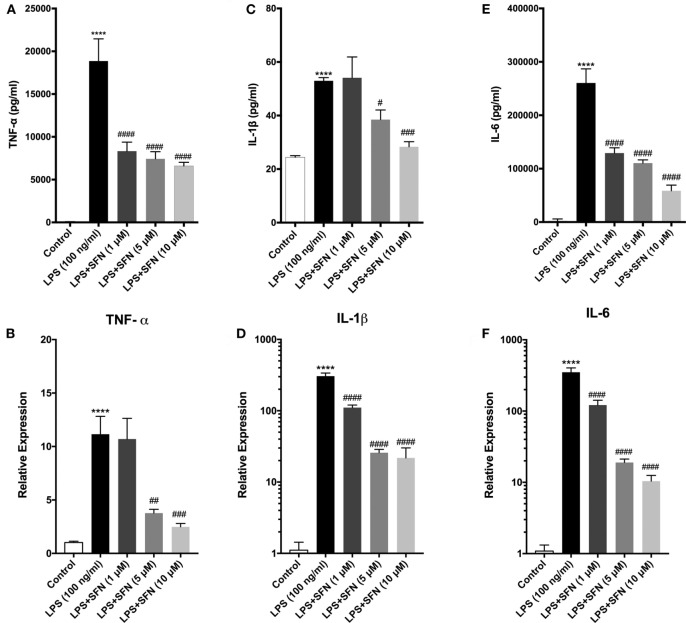
Sulforaphane (SFN) pretreatment reduced inflammatory cytokine production and secretion. N9 cells were treated with lipopolysaccharide (LPS) (100 ng/ml), then cytokine secretion for 24 h and cytokine mRNA expression for 6 h were analyzed. **(A,B)** Tumor necrosis factor-alpha (TNF-α), **(C,D)** interleukin-1 beta (IL-1β), and **(E,F)** interleukin-6 (IL-6) protein and mRNA levels were attenuated with SFN pretreatment (5 µM) 1 h prior to LPS treatment. The results are mean ± SEM, *n* = 5 (****p < 0.0001 compared with untreated control; ^#^*p* < 0.05, ^##^*p* < 0.01, ^###^*p* < 0.001, and ^####^*p* < 0.0001 compared with LPS treatment).

Sulforaphane exhibits its anti-inflammatory effect both at transcriptional and posttranscriptional levels. Therefore, the influence of SFN pretreatment on inflammatory cytokine production and secretion was examined by qPCR and ELISA method, respectively. To investigate the effect of SFN on pro-inflammatory cytokines (TNF-α, IL-1β, and IL-6) induced by LPS, we analyzed secreted cytokine levels upon LPS treatment with or without increasing concentrations of SFN (1–10 µM). SFN pretreatment reduced TNF-α, IL-1β, and IL-6 secretion in a concentration-dependent manner (Figures 3A,C,E). We analyzed mRNA expression levels of cytokines in N9 microglial cells as well. LPS treatment strongly induced mRNA expressions of TNF-α, IL-1β, and IL-6. Furthermore, pretreatment of SFN significantly attenuated mRNA expressions of these cytokines (Figures 3B,D,F).

### SFN Reduces LPS-Induced Oxidative Stress-Related Factors in N9 Microglial Cells

**Figure 4:**
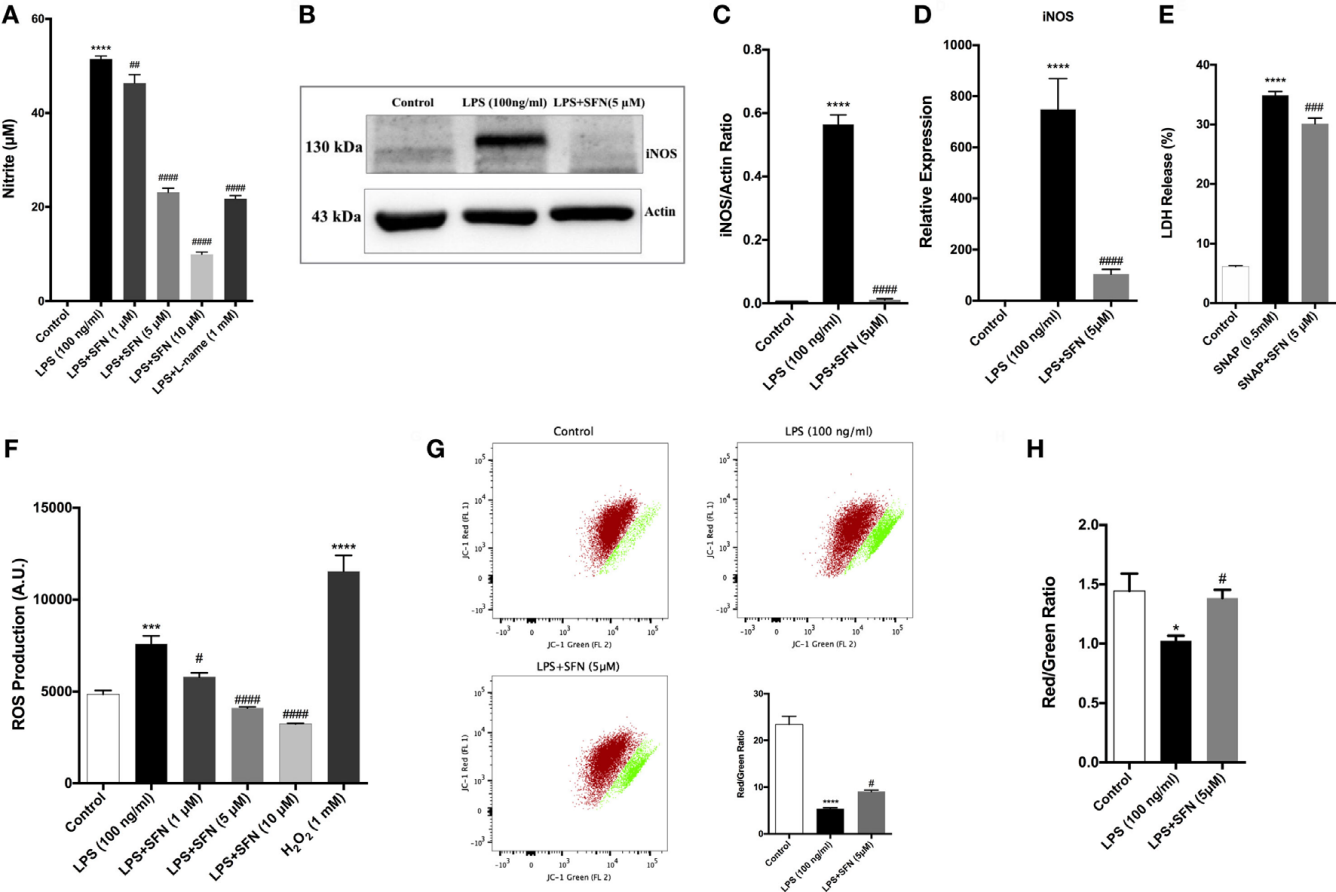
Sulforaphane (SFN) inhibited LPS-induced oxidative and nitrosative stress-related factors. N9 cells were treated with SFN (5 µM) for 1h prior to lipopolysaccharide (LPS, 100 ng/ml) treatment for 24 h, **(A)** nitrite levels were measured, **(B,C)** iNOS protein levels were detected by Western blot analysis using specific iNOS and β-actin antibodies, and **(D)** mRNA level of iNOS were analyzed with qPCR at 6 h after LPS treatment. **(E)** The effect of SFN on SNAP (NO-donor)-induced cell death was evaluated using lactate dehydrogenase (LDH) assay. **(F)** N9 cells were treated with SFN (5 µM) 6 h prior to LPS (100 ng/ml) treatment for 24 h and total reactive oxygen species (ROS) levels were measured. H_2_O_2_ was used as oxidative stress inducer. **(G)** Cells were treated with SFN (5 µM) 1 h prior to LPS (100 ng/ml) treatment for 24 h and mitochondrial membrane potential analysis were performed with using JC-1 dye with both flow cytometry and **(H)** enzyme-linked immunosorbent assay-based methods. SFN pretreatment decreases nitrite, iNOS, ROS production, and prevented impairment of mitochondrial membrane integrity. Also, SFN pretreatment exerted protection against NO-induced cell toxicity. The results are mean ± SE, *n* = 5 (*p < 0.05, ***p < 0.001, and ****p < 0.0001 compared with untreated control. ^#^*p* < 0.05, ^###^*p* < 0.001, and ^####^*p* < 0.0001 compared with LPS or H_2_O_2_ treatment).

Cytoprotective effect of SFN is related to its antioxidant effect. Therefore, the effect of SFN on various oxidative stress indicators (NO, iNOS, JC-1, DCF-DA) was also analyzed. To investigate whether SFN inhibited microglial LPS-induced nitrosative stress, we determined the level of nitrite with Griess reagent, and of iNOS expression by using qPCR and Western blot. We evaluated total nitrite release as an indicator of NO production. LPS significantly increased NO release in the culture medium; SFN pretreatment in N9 microglial cells significantly reduced NO production in a dose-dependent manner (Figure 4A). Next, we analyzed the effect of SFN treatment on iNOS mRNA and protein expression. The immunoblotting analysis clearly demonstrated the expression of iNOS proteins in LPS-treated N9 cells. SFN pretreatment prior to LPS stimulation significantly reduced iNOS protein expression (Figures 4B,C). qPCR analysis showed that iNOS mRNA expression was 500-fold higher in LPS-treated cells. On the other hand, SFN pretreatment downregulated LPS-induced iNOS mRNA expression (Figure 4D). In addition, we investigated whether SFN protects microglia against the NO-donor SNAP-induced cell death. SNAP treatment increased cell toxicity as a result of increased NO levels in N9 microglial cells, However, SFN pretreatment slightly decreased SNAP toxicity in N9 microglial cells (Figure 4E). Our results suggest that SFN may exert its inhibitory effect on SNAP-induced cell death through NO production in microglial cells.

We next measured total intracellular ROS levels using CH_2_-DCFDA. LPS-induced intracellular ROS levels were reduced with SFN pretreatment in a dose-dependent manner (Figure 4F). JC-1 staining was used to assess mitochondrial membrane potential. Pretreatment of microglia with SFN before LPS significantly maintained the mitochondrial membrane potential as exhibited by increased red/green fluorescence ratio of JC-1 in the surviving cells in comparison with microglia treated only with LPS (Figures 4G,H). The red/green fluorescence ratio was increased nearly 36% with SFN pretreatment when compared with LPS-treated microglia (Figures 4G,H).

### SFN Differentially Switches Activation States of Microglial Cells

**Figure 5:**
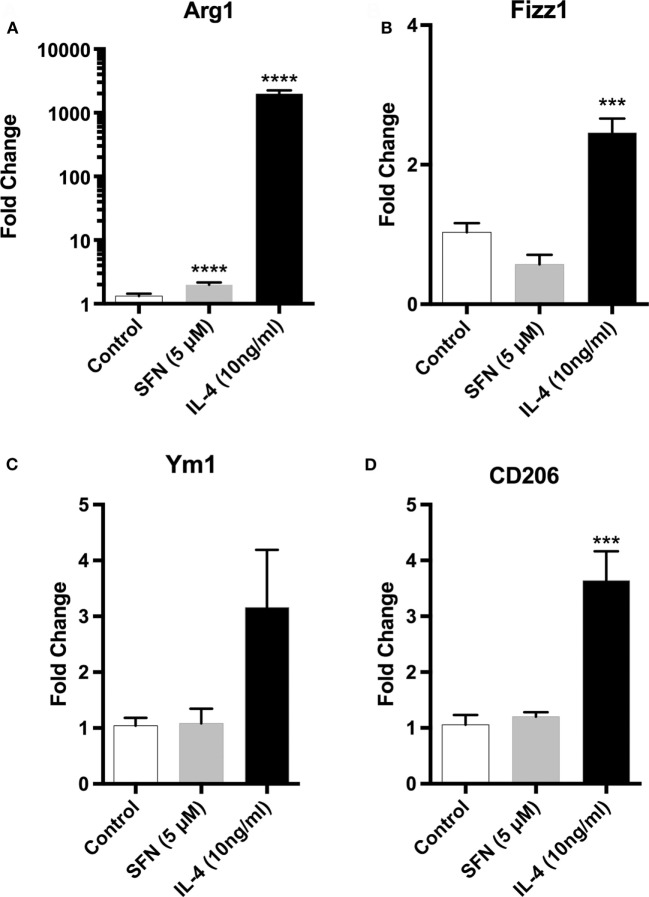
Sulforaphane (SFN) had no effect on M2 activation state of N9 cell. N9 cells were treated with SFN (5 µM) alone or IL-4 (10 ng/ml) alone for 24 h. For Arg1 expression analysis, treatment was 36 h. Microglial M2 status markers **(A)** Arg1, **(B)** Fizz1, **(C)** Ym1, and **(D)** CD206 were evaluated with qPCR. IL-4 was used as M2 polarization inducing agent. The results are mean ± SE, *n* = 5 (***p < 0.001 and ****p < 0.0001 compared with untreated control).

Since microglial cells show different activation states in the CNS, they can have both beneficial and harmful effects. Therefore, we investigated the effects of SFN on microglial activation states. First, we found that LPS treatment induced classical M1 status on N9 microglial cells (Figure 3). Furthermore, we analyzed the M2 state markers, namely Arg1, Ym1, Fizz1, and CD206, and we found that SFN did not cause polarization to M2 phenotype (Figure 5). On the other hand, we looked for the effects of SFN on Mox phenotype and we found that SFN treatment induced Mox phenotype related genes, Srxn1, Ho-1, and Gclc (Table 2).

**Table 2:** Expressions of SFN-induced nuclear factor erythroid 2-related factor 2 (Nrf2) target genes.

	Fold change	*p*-Value
*Nqo1*	10.69	0.009
*Ho-1*	7.50	0.009
*Gstp1*^a^	1.47	0.009
*Gclc*	2.35	0.009
*Srxn1*^b^	1.78	0.016

*^a^18 h*.

*^b^24 h*.

### SFN Inhibits NF-κB and AP-1 DNA-Binding Activities

**Figure 6:**
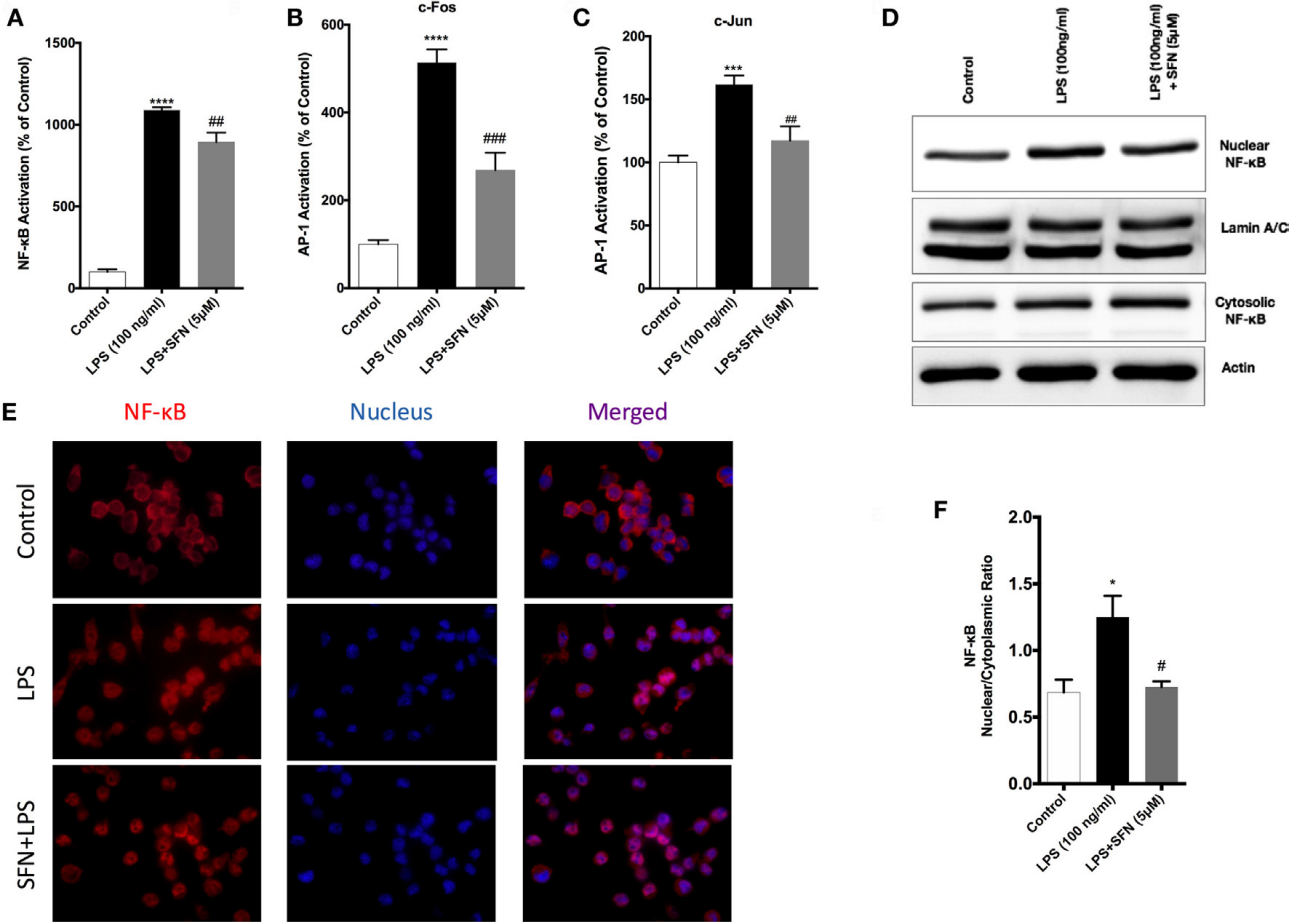
Inhibition of nuclear factor kappa-light-chain-enhancer of activated B cells (NF-κB) and activator protein 1 (AP-1) DNA-binding activity by sulforaphane (SFN). N9 cells were pretreated with SFN (5 µM) for 1 h. Then, cells were treated with lipopolysaccharide (LPS, 100 ng/ml) for 30 min. Markers **(A)** Arg1, **(B)** Fizz1, **(C)** Ym1, and **(D)** CD206 were detected TransAM, enzyme-linked immunosorbent assay-based DNA-binding assays. NF-κB translocation from cytoplasm to nucleus was evaluated with both **(D,E)** Western blot and **(F)** immunofluorescence imaging. LPS-induced NF-κB and AP-1 activities were reduced with SFN pretreatment. The results are mean ± SE, *n* = 5 (*p < 0.05, ***p < 0.001, and ****p < 0.0001 compared with untreated control. ^#^*p* < 0.05, ^##^*p* < 0.01, and ^###^*p* < 0.001 compared to LPS treatment alone).

To identify the mechanisms of SFN-mediated inhibition on LPS-induced microglial activation and cell death, we employed an ELISA-based method to determine the DNA-binding activities of transcription factors. LPS treatment induced NF-κB, AP-1 c-Fos, and c-Jun DNA-binding activity by 11-, 5-, and 1.5-fold, respectively. SFN pretreatment significantly attenuated LPS-induced NF-κB, c-Fos, and c-Jun subunits of AP-1 activities by 9-, 3-, and 1.2-fold, respectively (Figures 6A–C).

We also investigated the translocation of p65 subunit of NF-κB from the cytosol to the nucleus in microglia by Western blot and immunofluorescence staining. We found that LPS treatment induced NF-κB translocation from cytoplasm to nucleus about 1.4-fold and SFN pretreatment reduced translocation of NF-κB (Figures 6D,E). Furthermore, LPS treatment led to nuclear translocation of p65 subunit of NF-κB, as shown by strong NF-κB p65 staining in the nucleus and translocation of p65 from cytoplasm to nucleus was reduced with SFN pretreatment in N9 cells (Figure 6F).

### SFN Induces Nrf2 Activation and Expression of Its Target Genes

**Figure 7:**
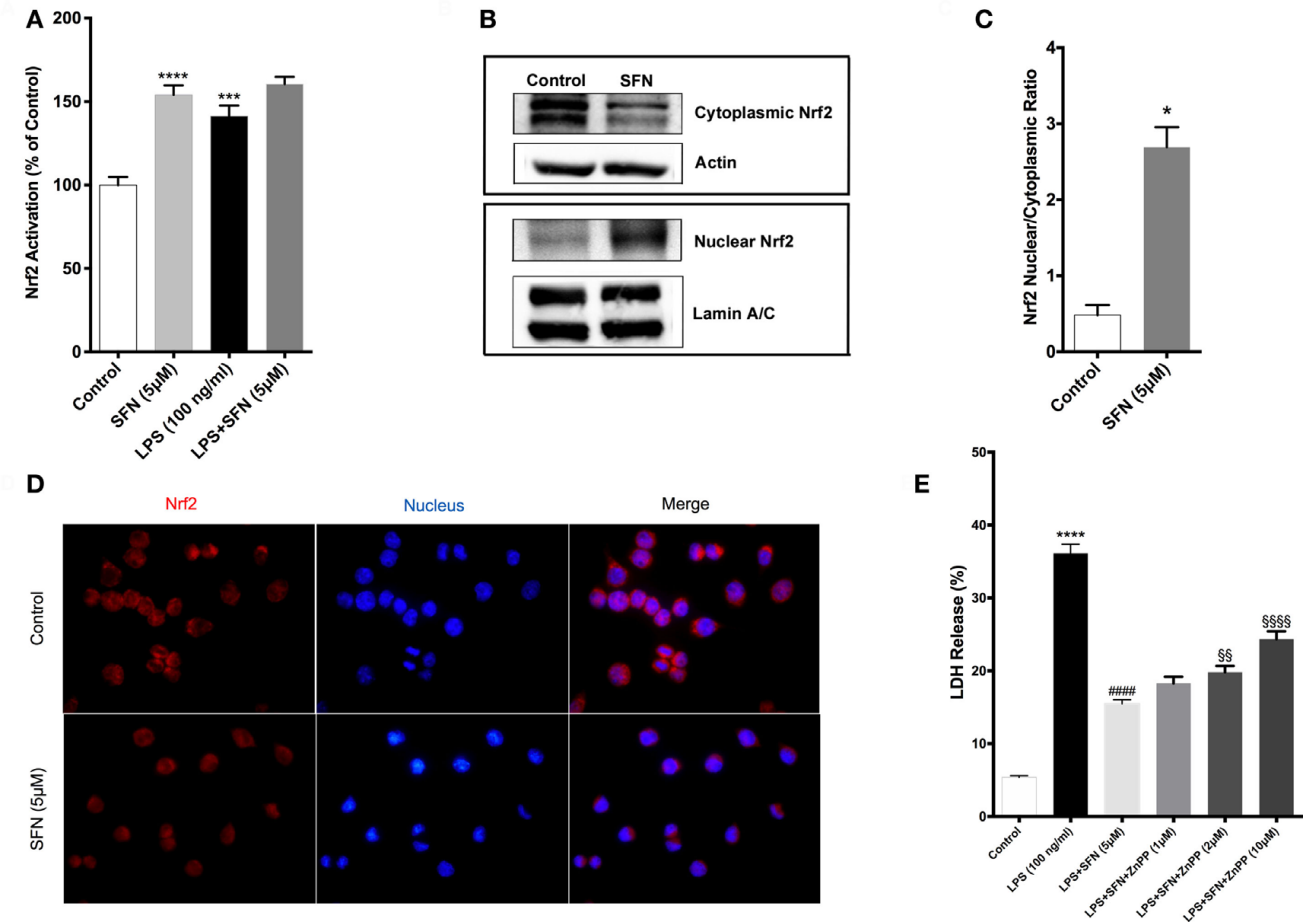
Sulforaphane (SFN) treatment induced nuclear factor erythroid 2-related factor 2 (Nrf2) activation in N9 cell line. N9 cells were treated with SFN (5 µM) alone and lipopolysaccharide (LPS) in the presence and absence of SFN for 3 h and nuclear extracts were prepared. **(A)** Nrf2 activation was evaluated with DNA-binding assay. **(B,C)** Nrf2 translocation was also evaluated with Western blot using Nrf2, β-actin, and Lamin A/C antibodies. **(D)** Immunofluorescence imaging of Nrf2 translocation from cytoplasm to nucleus cells was counterstained with Hoechst dye. **(E)** Cells were treated with Ho-1 inhibitor (ZnPP) for 2h prior to SFN and LPS treatment and cell death was assessed with lactate dehydrogenase (LDH) assay. The results are mean ± SE of three independent experiments. *p < 0.05, ***p < 0.001, and ****p < 0.0001 compared with untreated control. ^####^*p* < 0.0001 compared with LPS treatment and ^§§^*p* < 0.01, and ^§§§§^*p* < 0.0001 compared with LPS + SFN treatment.

Sulforaphane is already known as a potent inducer of Nrf2. Therefore, we examined the effects of SFN on Nrf2 activation by a DNA-binding assay. Consistent with previous studies (16), SFN alone significantly increased Nrf2 DNA-binding activity. Moreover, LPS alone also significantly increased binding activity of Nrf2 about 1.4-fold. Furthermore, LPS treatment with SFN pretreatment significantly increased Nrf2 activation further roughly 1.6-fold (Figure 7A). We also examined the effect of SFN on Nrf2 translocation in N9 cells. We found that treatment with SFN 5 µM for 3 h increased Nrf2 translocation from cytoplasm to nuclear about 2.5-fold, as shown by immunoblotting analysis (Figures 7B,C). Immunofluorescence staining also confirmed SFN treatment translocated Nrf2 from cytoplasm to nucleus (Figure 7D). We further demonstrated that chemical inhibition of Ho-1 with ZnPP resulted in significantly reduced cytoprotective effects of SFN upon LPS stimulation in a dose-dependent manner (Figure 7E).

We also used qPCR to analyze the effects of SFN on the expression levels of Nrf2 target genes, including Gclc, Gstp1, Ho-1, Srxn1, and Nqo1. As shown in Table S2 in Supplementary Material, Gclc, Ho-1, and Nqo1 mRNA expression levels were significantly upregulated upon SFN treatment for 6 h (2.35-, 7.50-, and 10.69-fold, respectively). Furthermore, Gstp1 mRNA levels were upregulated after 18 h of SFN treatment and also Srxn1 mRNA levels were significantly upregulated after 24 h SFN treatment (Table 2).

**Figure 8:**
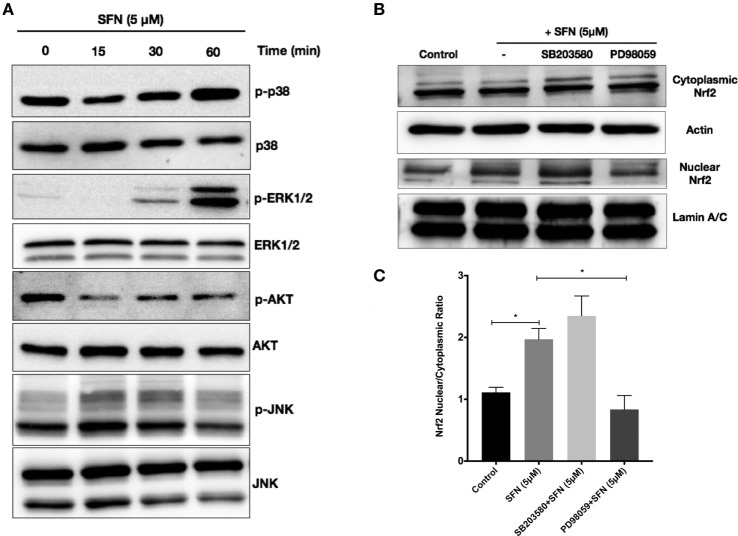
Sulforaphane (SFN) activated nuclear factor erythroid 2-related factor 2 (Nrf2) translocation through extracellular signal-regulated kinase 1/2 (ERK1/2) pathway. To evaluate the effect of SFN on p38, ERK1/2, PI3K/Akt, and JNK signaling pathways, N9 cells were treated with SFN (5 μM) for 15, 30, and 60 min and **(A)** p-p38 levels, p-ERK1/2, p-Akt, and p-JNK levels were examined with Western blot. Cells were treated with specific p38 (SB203580) and ERK1/2 (PD98059) inhibitors and Nrf2 translocation were analyzed with Western blot. **(B,C)** ERK1/2 inhibitor significantly inhibited Nrf2 translocation. The results are mean ± SE (SEM), *n* = 4 (*p < 0.05 and **p < 0.01 compared with start time).

We further attempted to determine upstream signaling pathways that SFN used to induce Nrf2 translocation. We treated N9 cells with SFN on different time points and analyzed activation of p38, extracellular signal-regulated kinase (ERK), JNK, and PI3K/Akt pathways. Interestingly, we found that phosphorylation of p38 and ERK1/2 was pronounced with SFN treatment for 60 min (Figure 8A). Encouraged by the observation of activation of p38 and ERK1/2, we investigated whether these pathways have influence on Nrf2 translocation upon SFN treatment. We showed that inhibition of ERK1/2 activation reduced translocation of Nrf2 from cytoplasm to nucleus (Figures 8B,C), suggesting that activity of ERK1/2 may be required for Nrf2 activation.

### Nrf2 Has a Key Role in the Protective, Antioxidant, and Anti-inflammatory Effects of SFN

**Figure 9:**
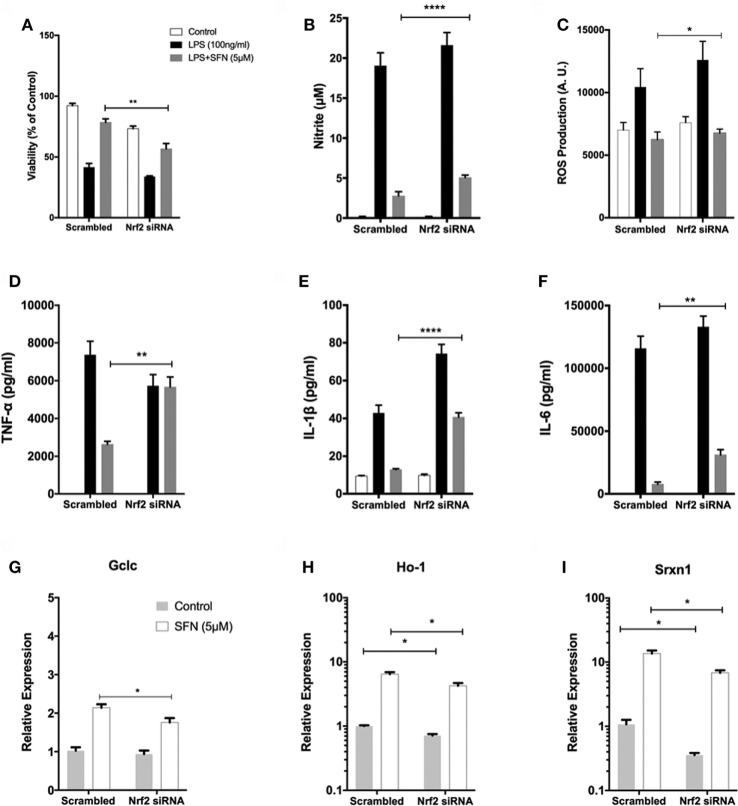
The role of nuclear factor erythroid 2-related factor 2 (Nrf2) transcription factor on protective effect of sulforaphane (SFN). N9 cells were transfected with negative control or Nrf2 siRNA for 24 h and then treated with SFN 1 h prior to LPS treatment. (A) Cell viability, (B) Nitrite levels, and (C) ROS levels were examined after Nrf2 transfection. Nrf2 knockdown abolished the protective effect of SFN on LPS-induced cell death and oxidative stress. (D) Tumor necrosis factor-alpha (TNF-α), (E) interleukin-1 beta (IL-1β), and (F) interleukin-6 (IL-6) levels were also measured after nuclear factor erythroid 2-related factor 2 (Nrf2) siRNA transfection. The anti-inflammatory effect of SFN was reversed by Nrf2 siRNA treatment. The effect of siRNA-mediated knockdown of Nrf2 mRNA was evaluated on Mox phenotype markers (G) glutamate cysteine ligase catalytic subunit (Gclc), (H) Ho-1, and (I) sulfiredoxin (Srxn-1) mRNA levels were quantified with qPCR. The results are mean ± SE, *n* = 5 (*p < 0.05, **p < 0.01, and ****p < 0.0001 compared with scrambled group).

To investigate the role of Nrf2 transcription factor on the protective effect of SFN, N9 cells were transiently transfected with scrambled (100 nM) and Nrf2 siRNA (100 nM) for 24 h. We examined whether siRNA-mediated Nrf2 knockdown would affect the protection by SFN pretreatment in N9 cells. Nrf2 siRNA transfection significantly reversed the protective effect of SFN on cell viability by lowering viability from 78 to 56% (Figure 9A). In addition, Nrf2 knockdown slightly reduced the inhibitory effects of SFN on both nitrite (Figure 9B) and ROS levels (Figure 9C). Next, we questioned whether the anti-inflammatory effect of SFN was dependent on Nrf2. To this end, we measured pro-inflammatory cytokine secretion levels (TNF-α, IL-1β, and IL-6) in siRNA transfected N9 cells. We found that siRNA-mediated Nrf2 knockdown significantly reversed suppression of TNF-α secretion levels approximately 2.15-fold by SFN pretreatment (Figure 9D). In addition, Nrf2 knockdown significantly reversed IL-1β secretion around 3.15-fold when compared with scrambled group (Figure 9E). Similarly, IL-6 secretion levels were significantly increased about 4.5-fold upon Nrf2 knockdown (Figure 9F). In the next step, we evaluated Nrf2 dependency in Mox phenotype in N9 cells. After Nrf2 knockdown, levels of SFN-induced Mox phenotype markers were reduced (Figures 9G–I).

### Pro-inflammatory miRNA Levels Are Attenuated with SFN Treatment

**Figure 10:**
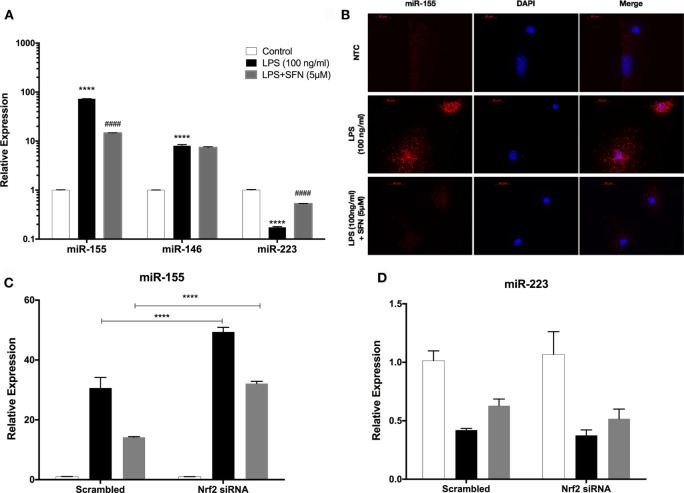
Sulforaphane (SFN) modulated miR-155 and miR-223 expression in N9 cells. N9 cells were treated with SFN (5 µM) prior to lipopolysaccharide (LPS, 100 ng/ml) treatment for 24 h. **(A)** miR-155, miR-146, and miR-223 levels were analyzed with qPCR. **(B)** FISH analysis of miR-155 in primary mouse microglial (PMG) cells. After nuclear factor erythroid 2-related factor 2 (Nrf2) siRNA transfection **(C)** miR-155, and **(D)** miR-223 levels were measured. Inhibitory effect of SFN on miR-155 expression was reversed with Nrf2 siRNA transfection. The results are mean ± SE, *n* = 5 (****p < 0.0001 compared with untreated control. ^####^*p* < 0.0001 compared with LPS treatment. In figure 9C, ****p < 0.0001 compared with scrambled group).

miR-155, miR-146a, and miR-223 are known as immune miRNAs in brain and their expression levels in brain are associated with brain injury and neurodegenerative diseases (35, 36). We, therefore, investigated the effects of SFN on the levels of these pro-inflammatory miRNAs. We used LPS treatment to induce expression of these miRNAs. We found that LPS treatment significantly increased miR-155, miR-146a, and miR-223 expression levels in N9 cells (Figure 10A). Furthermore, SFN pretreatment significantly attenuated increased levels of miR-155 by LPS and increased downregulated miR-223 by LPS in microglia (Figure 10A). ISH also confirmed the effect of SFN on reducing LPS-induced miR-155 expression in primary microglial cells (Figure 10B). Furthermore, we hypothesized that the inhibitory effects of SFN on these miRNAs might be Nrf2-dependent. Therefore, we analyzed the expression levels of these miRNAs after siRNA-mediated Nrf2 knockdown. We found that the inhibitory effects of SFN on miR-155 are substantially reversed after Nrf2 knockdown (Figure 10C). However, miR-223 expression did not change (Figure 10D).

### SFN Pretreatment Could Protect Neurons from Activated Microglial Toxicity

**Figure 11:**
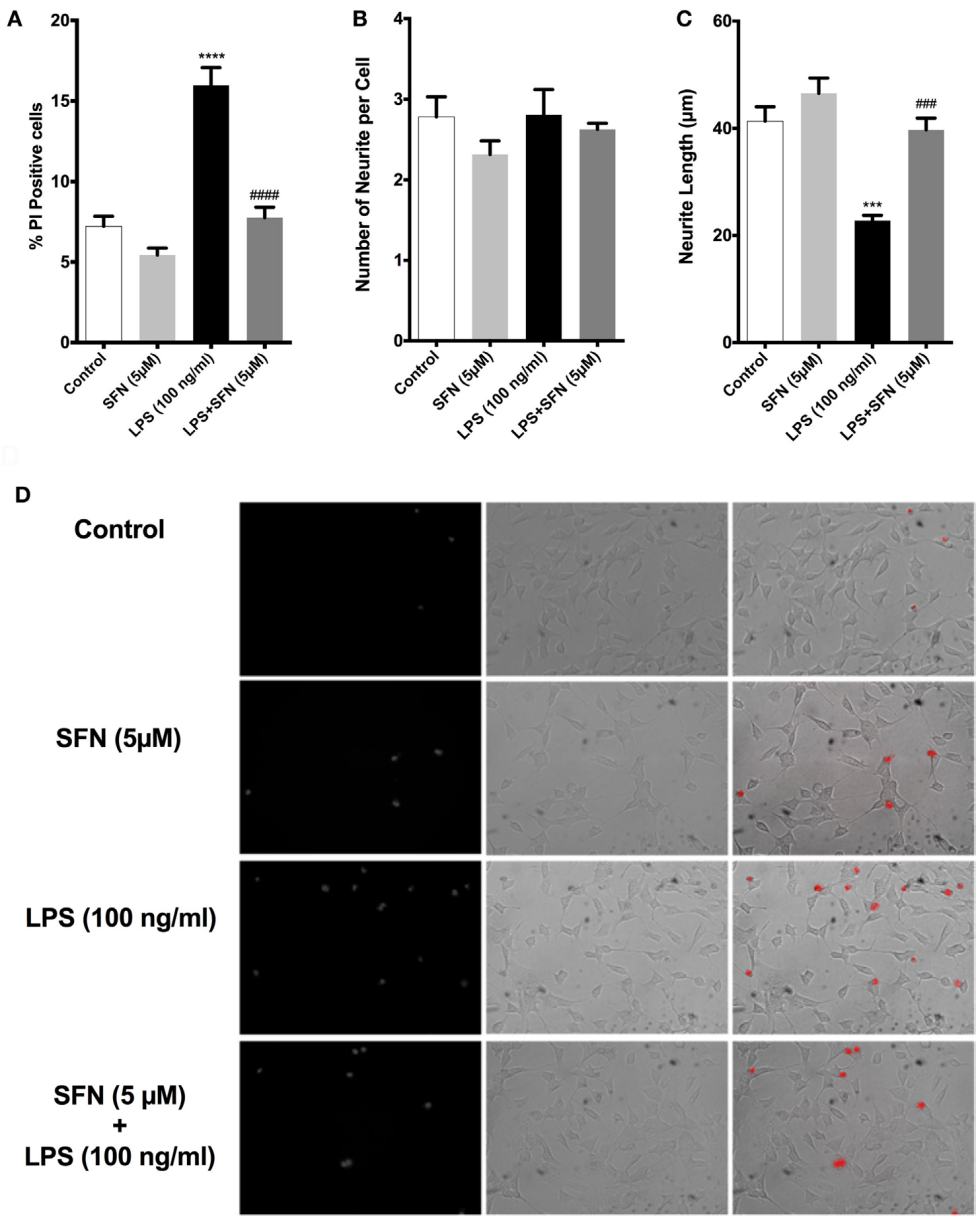
Sulforaphane (SFN) protected SH-SY5Y cells against microglial activation in coculture system. **(A)** Cell death, **(B)** neurite number, and **(C)** neurite length of SH-SY5Y cells were measured after coculturing with N9 cells. **(D)** Representative images of SH-SY5Y cells in coculture system. The results are mean ± SE, *n* = 5 (***p < 0.001 and ****p < 0.0001 compared with untreated control. ^###^*p* < 0.001 and ^####^*p* < 0.0001 compared with lipopolysaccharide (LPS) treatment).

We used both a coculture system and conditioned medium experimental designs to investigate whether SFN could reduce the detrimental properties of activated microglial cells. In both experimental setups, LPS-induced activation of N9 cells resulted in a significant increase in neuronal cell death and a significant decrease in neurite length (Figure 11). On the other hand, SFN pretreated N9 cells and their supernatants had lower toxicity on SH-SY5Y cells. Conditioned media from LPS activated N9 cells decreased number of neurites in SH-SY5Y cells. Neurite length was improved in both experimental setups. Neurite number of SH-SY5Y cells was increased by SFN conditioned media (Figure S1 in Supplementary Material). These results suggest that SFN pretreatment could significantly reduce the neurotoxic effects of activated microglial cells.

## Discussion

In the present study, we showed that SFN protected murine microglial cells from LPS-induced cell death. Functional studies with Nrf2 siRNA showed that the cytoprotective, antioxidant, and anti-inflammatory effects of SFN are Nrf2 activation dependent. Our second main finding is that SFN attenuates microglial polarization. It inhibits M1 phenotype-specific gene expression and induces Mox phenotype-specific gene expression. Furthermore, we also found that SFN caused downregulation of LPS-induced expression of miR-155 and upregulation of miR-223 expression inhibited by LPS.

Lipopolysaccharide binds to toll-like receptor 4 (TLR4) on the cell surface, thereby activating a cascade of pro-inflammatory responses through MAPKs (37). Through its downstream adaptor protein myeloid differentiation primary response gene 88, TLR4 pathway activates NF-κB and AP-1 transcription factors to express pro-inflammatory mediators (38). LPS may also induce cell death through the MAPK pathway and NF-κB transcription factor. Oxidative and nitrosative stress, inflammation, and mitochondrial dysfunction intricately interplay to induce cell death (39). We found that LPS treatment leads to both apoptotic and necrotic cell death in N9 microglia. Previous studies demonstrated that LPS stimulation causes intrinsic apoptosis in microglial cells (40, 41). LPS-induced cell death of microglia occurs through either NO- or Caspase-11-dependent fashion, both of which then converge on executioner Caspase-3 (42). Using a set of apoptosis assays, we determined that LPS-induced microglial cell death occurs through intrinsic apoptotic pathway and in both NO- and caspase-11-dependent fashion. In consistent with previous studies (40, 42), we found that LPS-induced cell death is partly NO-dependent manner. In our hands, both caspase-3 and caspase-11 activities increased by LPS stimulation suggesting that caspase-11-dependent apoptotic cell death is also involved in microglial cell death.

We showed that SFN pretreatment attenuated LPS-induced apoptotic and necrotic cell death of N9 cells. SFN treatment also ameliorated microglial activation-induced neuronal cell death in both coculture and conditioned medium experiments. SFN exerts cytoprotective effects either directly or *via* Nrf2-mediated inhibition of NF-κB (29, 43). As shown by Beyaert and his colleagues in murine macrophage cell line, Caspase-11 is one of the NF-κB target genes (44) and autoproteolytically converts to its mature form. Caspase-11, a crucial initiator caspase, activates downstream effector caspases, including caspase-3 and caspase-1 and leads to both apoptotic and pyroptotic cell death (33, 45). We found that SFN pretreatment decreased LPS-induced caspase-11 activity and DNA binding of NF-κB. It has been implicated that LPS-induced NO-dependent cell death is also started by NF-κB activation (46). Thus, it seems that SFN inhibits both intrinsic apoptotic cell death pathways (NO- and caspase-11 dependent) converging on caspase-3 *via* the suppression of NF-κB transcription factor.

In our study, we showed that SFN attenuated LPS-induced ROS accumulation in a dose-dependent manner in N9 microglia. The cytoprotective, anti-inflammatory, and antioxidant role of SFN can be dependent on the activation of Nrf2 transcription factor. SFN upregulated Nrf2 target gene expression levels in mice models and microglial cell lines (21, 22). We found that SFN activates Nrf2- and siRNA-mediated knockdown of Nrf2 reversed the effect of SFN on LPS-induced ROS, RNS, and pro-inflammatory cytokine production and cell viability suggesting that they are at least in part, Nrf2-dependent.

However, knockdown of Nrf2 differentially affected various actions of SFN. Nrf2 inhibition moderately reversed the cytoprotective effect of SFN. While antioxidant and anti-nitrosative effects of SFN modestly, but significantly decreased, secreted concentrations of pro-inflammatory cytokines apparently increased in Nrf2 knockdown conditions. Elimination of SFN-mediated anti-inflammatory effect of by Nrf2 knockdown may result from upregulation of pro-inflammatory mediators through activation of redox sensitive transcription factors NF-κB and AP-1 in oxidative environment and removal of Nrf2-mediated NF-κB inhibition. Since activation of Nrf2 by SFN exerts dissociation of Keap1 and upregulation Ho-1 to inhibit NF-κB pathway (43). SFN exerts its cytoprotective effect *via* activation of Nrf2 transcription factor and subsequent upregulation of Ho-1, through Mox phenotype switch. We found that ZnPP, a chemical inhibitor of Ho-1, dose-dependently abolished the cytoprotective effect of SFN upon LPS-induced cell death. Based on these findings, SFN has cytoprotective effect on AICD in N9 microglial cells in partly Nrf2- and Ho-1-dependent manner. However, the mechanism of the activation of Nrf2 by SFN is still unclear. Previous studies reported that ERK1/2, JNK, P38, and PI3K/Akt pathways might be involved in Nrf2 activation by SFN in various cell types (47). We found that the ERK1/2 inhibitor achieved inhibition of Nrf2 translocation. This finding suggested that ERK1/2 was involved in Nrf2 activation.

Microglial cells have the capacity to switch their polarization phenotypes and persistent predominance of M1 activation state has been associated with many neurodegenerative diseases and aging (48). M1 microglia initiate innate immune responses in CNS and release pro-inflammatory cytokines and iNOS, while M2 phenotype provides tissue repair and resolution of inflammation (49, 50). Here, we showed that SFN inhibited LPS-induced M1 phenotype of N9 microglia and it failed to switch to M2 phenotype. In addition, another microglial activation state has been defined as Mox phenotype (10). Hua et al. have recently shown that murine primary microglial cells can also be polarized into Mox phenotype with combined use of α7 cholinergic receptor agonist AR-R 17779 and LPS (10). We also found that SFN alone upregulated mRNA expression levels of Mox markers, namely Ho-1, Srxn1, and Gclc, in murine N9 microglial cell line. Suppression of Mox phenotype with Ho-1 inhibitor reversed cytoprotective effect of SFN in N9 cells.

miR-155 has been shown to have critical roles in inflammation, such as involvement in microglial activation (34). miR-155 is processed from exon 2 of a gene B-cell integration cluster located on chromosome 21, by AP-1 through c-Jun N-terminal kinase activation and NF-κB (51, 52) and has also been identified and characterized in monocyte and macrophage in response to TLR ligands, such as LPS (53). As a result of our PCR and ISH analyses, we demonstrated that SFN inhibited the expression of miR-155 in response to LPS. Inhibition of Nrf2 also resulted in attenuation of protective effect of SFN, which implies the regulatory role of Nrf2 in miR-155 regulatory network. However, the recovering effect of SFN on miR-223 expression is Nrf2-dependent. miR-223 is a posttranscriptional regulator of inflammasome related protein NLRP3 (54). This is the first demonstration that microglial miR-223 expression is regulated by SFN.

In the present study, we used murine N9 immortalized microglial cells in addition to primary murine microglia. This cell line is well characterized and widely used (55). *In vitro* studies are valuable and indispensable for well-designed animal experiments. The advantages of cell line cultures are their ease of maintenance, homogeneity, high yield, cost-effectiveness, time saving, and avoidance of animal experimentation. In addition, cell lines are suitable for high-throughput analyses such as in cell toxicity and drug discovery studies. One limitation of our study is the absence of *in vivo* studies. Although, upon activation by immune stimuli, microglial cell lines exhibit very similar cellular responses to primary microglia, some differences have been reported in terms of gene and protein profiles (56). Extrapolation of *in vitro* findings to *in vivo* results is still challenging. In spite of ethical concerns, animal studies are eventually required before clinical trials (57). To date, only one *in vivo* study reported that SFN showed beneficial effects against LPS administration in mice microglia (22). Yet, the effects of SFN on microglial phenotypic alterations and cell death have not been investigated in that study. Our findings should also be confirmed with human microglia.

Since SFN has a lipophilic and low molecular weight nature as a food-derived non-nutrient molecule, it easily crosses BBB to act as a nutrigenomic activator of Nrf2 (17). SFN attenuated microglial cell death by selectively inhibiting ROS- and RNS-producing NF*-*κB and AP-1 transcription factors. SFN also activates antioxidant Nrf2 transcription factor to combat against oxidative environment upon LPS exposure. SFN has two-sided effects on activation states, i.e., it suppresses LPS-mediated M1 activation and converts it to Mox phenotype to prevent from inflammation, redox imbalance, and cytotoxicity. We can also conclude that suppression of LPS-induced cell death and M1 polarization and induction of Mox polarization mediate cytoprotective, anti-inflammatory, and antioxidant effects of SFN. Furthermore, our findings indicate that SFN also alleviated LPS-induced expression of pro-inflammatory miRNA miR-155.

Finally, SFN inhibited microglia-mediated neurotoxicity as shown by conditioned medium and coculture experiments. Our results suggest that the cytoprotective, anti-inflammatory, and antioxidant effects of SFN are mediated by ERK1/2-Nrf2. Thus, SFN could be a promising drug candidate based on its therapeutic potential for modulating inflammatory responses and cell survival in CNS.

## Author Contributions

EE, KT, KG, and SG design the study. EE, KUT, KBI, and BT designed and performed the experiments. EE, KUT, KG, and SG analyzed and interpreted the data. EE, KUT, KG, and SG wrote the manuscript. EE, KUT, KBI, BT, KG, and SG read and approved final manuscript.

## Conflict of Interest Statement

The authors declare that the research was conducted in the absence of any commercial or financial relationships that could be construed as a potential conflict of interest.

## Supplementary Material

Figure S1 Conditioned media from Sulforaphane (SFN)-pretreated N9 cells protected SH-SY5Y cells against microglial cell activation. N9 cells were pretreated with or without SFN (5 µM) and then, LPS (100 ng/ml) treatment for 24 h and culture media were taken as conditioned media. After culturing SH-SY5Y cells with conditioned media **(A)** cell death, **(B)** neurite number, and **(C)** neurite length were assessed. **(D)** Representative images of conditioned media treated SH-SY5Y cells. The results are mean ± SE, *n* = 5 (**p* < 0.05 and ***p* < 0.01 compared with untreated control; ^##^*p* < 0.01 compared with LPS treatment).
