# Supplementary material for: Sulforaphane Inhibits Lipopolysaccharide-Induced Inflammation, Cytotoxicity, Oxidative Stress, and miR-155 Expression and Switches to Mox Phenotype through Activating Extracellular Signal-Regulated Kinase 1/2–Nuclear Factor Erythroid 2-Related Factor 2/Antioxidant Response Element Pathway in Murine Microglial Cells
Source: Front Immunol. 2018 Jan 23;9:36. doi: 10.3389/fimmu.2018.00036 (PMC5787131; doi:10.3389/fimmu.2018.00036)
Supplement: Supplementary file 1 [file Table_1.DOCX]

**Supplementary Table 1:** Antibody List

| **Antibody** | **Dilution** | **Catalog Number** | **Provider** |
| --- | --- | --- | --- |
| Akt | 1:1000 | 4685 | Cell Signaling, USA |
| Anti-rabbit IgG HRP | 1:3000 | 7074 | Cell Signaling, USA |
| β-actin | 1:10000 | ab8227 | Abcam, USA |
| Donkey Anti-rabbit Alexa Fluor 594 | 1:2000 | 711-585-152 | Jackson Immunoresearch, USA |
| ERK1/2 | 1:500 | sc-93 | Santa Cruz, USA |
| iNOS | 1:1000 | 13120 | Cell Signaling, USA |
| JNK | 1:1000 | 9252 | Cell Signaling, USA |
| Lamin A/C | 1:1000 | sc-20681 | Santa Cruz, USA |
| NF-κB p65 | 1:500 | sc-372 | Santa Cruz, USA |
| Nrf2 | 1:500 | sc-722 | Santa Cruz, USA |
| p38 | 1:500 | sc-7149 | Santa Cruz, USA |
| phospho-p38 | 1:1000 | 4511 | Cell Signaling, USA |
| phospho-Akt | 1:1000 | 4058 | Cell Signaling, USA |
| phospho-ERK1/2 | 1:2000 | 4370 | Cell Signaling, USA |
| phospho-JNK | 1:1000 | 4668 | Cell Signaling, USA |
