# Supplementary material for: Sulforaphane Inhibits Lipopolysaccharide-Induced Inflammation, Cytotoxicity, Oxidative Stress, and miR-155 Expression and Switches to Mox Phenotype through Activating Extracellular Signal-Regulated Kinase 1/2–Nuclear Factor Erythroid 2-Related Factor 2/Antioxidant Response Element Pathway in Murine Microglial Cells
Source: Front Immunol. 2018 Jan 23;9:36. doi: 10.3389/fimmu.2018.00036 (PMC5787131; doi:10.3389/fimmu.2018.00036)
Supplement: Supplementary file 2 [file Table_2.DOCX]

**Supplementary Table 2:** Primer List

| **Gene** | **Forward Sequence (5’🡪3’)** | **Reverse Sequence (5’🡪3’)** |
| --- | --- | --- |
| Arg1 | CAGAAGAATGGAAGAGTC | CAGATATGCAGGGAGTCA |
| CD206 | CAGGTGTGGGCTCAGGTAGT | TGTGGTGAGCTGAAAGGTGA |
| Fizz1 | AGACTTGCGTGACTATGAAGCATTG | GGCCCATCTGTTCATAGTCTTGA |
| G3PDH | ACCACAGTCCATGCCATCAC | TCCACCCTGTTGCTGTA |
| Gclc | GGCTCTCTGCACCATCAC | TCTGACACGTAGCCTCGG |
| Gstp1 | CGGCAAATATGTCACCCTCA | GTTCACATGTTCCGGGGAGG |
| Ho-1 | GAGACGGCTTCAAGCTGGTGATG | GTTGAGCAGGACGCAGTCTTGG |
| IL-1β | CACAGCAGCACATCAACAAG | GTGCTCATGTCCTCATCCTG |
| IL-6 | GTGGTATCCTCTGTGAAGTCT | AAGAGCTTCCAGCCAGTTGCC |
| iNOS | CCACAATAGTACAATACTACTTGG | ACGAGGTGTTCAGCGTGCTCCACG |
| Nqo1 | GCCTAGCACAAGTACCACTCTTGGTC | CTGAGGCAGGAGAATTGCTGGAACC |
| Nrf2 | AGATTCACAGGCCTTTCTCG | CAGCTCTCCCTACCGTTGAG |
| Srxn1 | GAAGAGGTATGGGGCTAC | GCAGCCCCCAAAGGAATA |
| Tnf-α | GTGCCACTTCATACCAGGAGAA | TCACAGACGAATGACTCCAA |
| Ym1 | AGAAGGGAGTTTCAAACCTGGT | GTCTTGCTCATGTGTGTAAGTGA |
