# Supplementary figures and images for: Sulforaphane Inhibits Lipopolysaccharide-Induced Inflammation, Cytotoxicity, Oxidative Stress, and miR-155 Expression and Switches to Mox Phenotype through Activating Extracellular Signal-Regulated Kinase 1/2–Nuclear Factor Erythroid 2-Related Factor 2/Antioxidant Response Element Pathway in Murine Microglial Cells
Source: Front Immunol. 2018 Jan 23;9:36. doi: 10.3389/fimmu.2018.00036 (PMC5787131; doi:10.3389/fimmu.2018.00036)

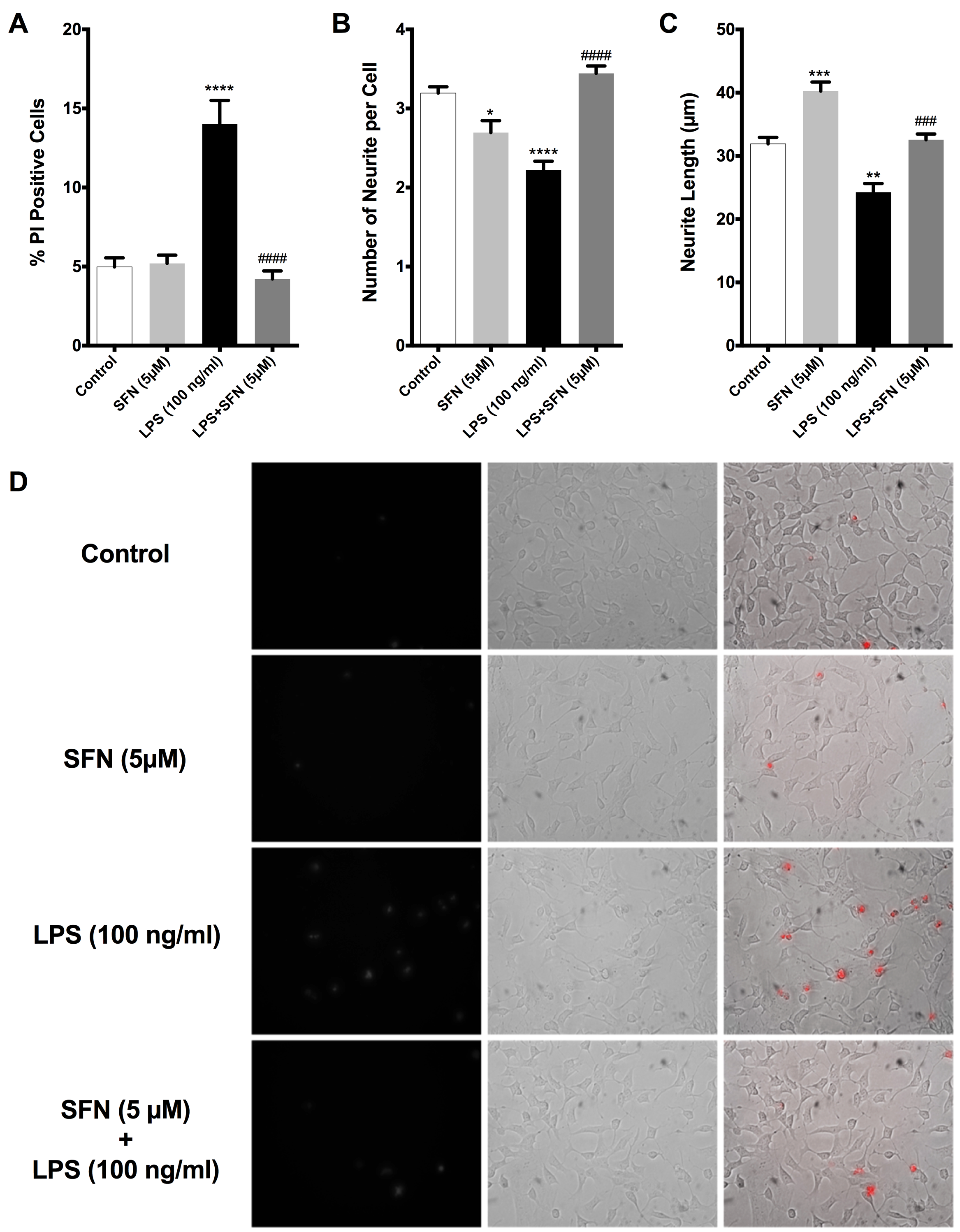

Supplement: Figure S1 — Conditioned media from Sulforaphane (SFN)-pretreated N9 cells protected SH-SY5Y cells against microglial cell activation. N9 cells were pretreated with or without SFN (5 µM) and then, LPS (100 ng/ml) treatment for 24 h and culture media were taken as conditioned media. After culturing SH-SY5Y cells with conditioned media (A) cell death, (B) neurite number, and (C) neurite length were assessed. (D) Representative images of conditioned media treated SH-SY5Y cells. The results are mean ± SE, n = 5 (*p < 0.05 and **p < 0.01 compared with untreated control; ##p < 0.01 compared with LPS treatment). [file Image_1.TIFF]
